# A vision for the innovative study of fungal biology in China: Presidential address

**DOI:** 10.1080/21501203.2015.1026112

**Published:** 2015-03-20

**Authors:** Chengshu Wang

**Affiliations:** Institute of Plant Physiology and Ecology, Shanghai Institutes for Biological Sciences, Chinese Academy of Sciences, Shanghai200032, China

**Keywords:** presidential address, fungal biology, innovative research, scientific version

## Abstract

I am proud to be elected as the sixth president of the Mycological Society of China, and highly pleased to have a chance to share my personal opinion here with my fellow mycologists and students regarding the innovative performance of fungal biology studies in China. A stepwise buildup of knowledge and sharp scientific vision is the prerequisite for innovative studies. Taken together with the most advanced techniques and elegant experimental designs, the scholars would have a better chance to acquire novel and conceptual results rather than the “me too” stories by focusing on the mechanisms related with fungal unique biology.

I am honored and proud to speak with you as the sixth President of the Mycological Society of China (MSC). MSC was divided from the Botanical Society of China and found in May 1993. The Society currently has over 3000 registered members involved in the studies from the systematics, genetics, genomics to applied biology of the oomycetes, myxomycetes, lichen-forming fungi, mycorrhiza, mushrooms to plant and animal pathogenic fungi. A few research groups in China are working with the model fungi such as *Neurospora crassa, Aspergillus nidulans, Saccharyomyces cerevisae* as well as the model plant- and mammalian-pathogenic fungi like *Magnaporthe oryzae, Fusarium graminearum, Candida albicans, A. fumigatus* and *Cryptococcus neoformans*. Innovative works have been published by these groups from the mechanisms of epigenetic controls of development to pathogenesis (e.g., Zhou et al. 2012, 2013; Xie et al. 2013; Tao et al. 2014). Otherwise, many more groups are conducting the taxonomy/systematics and applied studies of fungal biology. Relative to the creative works on fungal biology contributed from the scientists from the Western countries, we agree that the breakthrough works published by the Chinese mycologists are still rare. Along with the massive decoding of the non-model fungal genomes in China (e.g., An et al. 2010; Zheng et al. 2011; Chen et al. 2012; Hu et al. 2013; Lai et al. 2014; Zhang et al. 2014; Wang et al. 2015) and the successful application of *Agrobacterium*-mediated fungal transformation (even the newly emerged CRISPR/CAS9 system) technique etc. (DiCarlo et al. 2013), I would like to share the vision with you for the future creative and innovative studies of fungal biology by working on the non-model fungal species.

Recognition of new fungal species, and phylogenetic and or phylogenomic studies are the basic and enduring works for elucidation of fungal-tree-of-life. In recent years, however, the concepts of population genomics, pan-genomics and GWAS (genome-wide association study) have been deployed to explore the genomic features or patterns of evolutionary trajectory, environmental adaptation, co-evolution, domestication and rate of evolution of a particular fungal lineage (Palma-Guerrero et al. 2013; Hu et al. 2014; Lanfear et al. 2014). For innovative studies of fungal taxonomy and systematics, my vision is that additional care should be considered during the collection of fungal samples to maximally represent a diverse array of geographic and\or host origins for generating a monograph on particular lineage(s). This is also applicable for the design of *de novo* genome sequencing of non-model fungal species (Ellegren 2014; Gladieux et al. 2014). Similar to the reports of a handful of new fungal species, genome sequencing and comparative genomic studies of a single or a few taxonomically or biologically related fungal species are still publishable, however, the novel and conceptual conclusions usually require the solid supports from massive analyses of a large population or community of fungal lineages.

For the species from lichen-forming fungi to oomycetes, genetic transformation is no longer the technical bottleneck for functional genomic studies of non-model fungal species. However, many “me too” studies are being conducted by the Chinese mycological groups, for example, the deletions and characterizations of the highly conserved genes involved in conidiation, mycelium development, sexuality or stress responses etc. that have been well documented in model species like *Neurospora, Aspergillus* and *Saccharyomyces*. These works are also publishable and particularly favored by the students to obtain the mutant strains with visible phenotypes. It is generally understandable for the groups to start “molecular” works on non-model fungi. The literature reviews also indicate that the tenet of ortholog conjecture is not always applicable, i.e., functional divergence of orthologous genes evident in different fungal species (Huang et al. 2015). We well understand that each species is biologically unique. Thus, novel and interesting works are welcomed only for those functional studies of the genes or pathways associated with fungal unique biology. Indeed, these works are highly challenging and frequently abortive. Except for functional redundancy, the phenotypes are always not visible after deletion of these genes, and thus the problems for designing the follow-up experiments. Risk and chance coexist. The successes of this type of works are always interesting and novel. Otherwise, research focuses should at least be paid for the mechanisms of host infection/interaction for pathogenic fungi, establishment of symbiotic relationships for mycorrhiza and lichen-forming fungi, mating and fruiting-body development for mushrooms, adaptation for fungi from extreme environments, bioactive metabolite biosynthesis for medicinal fungi as well as the engineering for maximum production of enzymes or metabolites in industrial fungi.

Many things matter for conducting innovative studies and generating creative results. Besides the stepwise buildup of sharp scientific visions, what I mostly want to share the experience with you is to better present your results. Based on my editorial experiences for international journals, I find that the qualities of many submissions from Chinese mycologists/scientists are suffering from the English-writing and presentation issues. Grammar issues can now be solved by scientific editing services. However, the company will usually not help for the structure issues of data presentation and inconsistency/non-relevance of introduction and result discussion, which could significantly downgrade the quality and thereby the novelty of a work. Indeed, it is a common issue for non-native English speaking scientists worldwide; however, the problems should be well appreciated by the Chinese scientists to submit their works with real for-the-first-time findings.

## Disclosure statement

No potential conflict of interest was reported by the author.
